# Somalia cannot outbreak-respond its way out of a climate crisis: Building health sovereignty througone health

**DOI:** 10.1016/j.onehlt.2026.101569

**Published:** 2026-09-11

**Authors:** Ayuub Ahmed Osman, Maryan Ali Adam, Abdisamad Abdihakim Aadam, Ilyas Abdullahi Khalif, Mohamed Abdijabar Mohamed, Maryamo Maxamed Cali

**Affiliations:** Faculty of Health Sciences and Tropical Medicine, Somali National University, Mogadishu, Somalia; Banadir Research, Consultancy & Evaluation Center, Mogadishu, Somalia

**Keywords:** Somalia, Climate change, One Health, Health security, Health sovereignty

## Abstract

Somalia faces recurrent climate shocks that intensify food insecurity, displacement, service disruption, and infectious-disease risk. Although outbreak response remains essential, repeated emergency action cannot address the structural conditions that sustain vulnerability. This commentary argues for a shift from reactive outbreak control toward climate-resilient health security grounded in health sovereignty and One Health. We propose integrated climate–One Health early warning, stronger surveillance, resilient WASH and primary healthcare, anticipatory financing, and locally led capacity to prevent climate-sensitive health emergencies before they escalate.

Dear Editor,

Somalia's health emergency is now defined by a stark paradox: the country repeatedly mobilizes to contain outbreaks, yet the climatic and socioeconomic drivers of vulnerability continue to intensify. Recent estimates indicate that around 5 million people will require health assistance in 2026, while 6.5 million people are projected to face acute food insecurity and about 1.84 million children aged 6–59 months acute malnutrition. These are not separate crises. They are interlocking conditions that regenerate epidemic risk. Outbreak response remains indispensable, but it is insufficient when droughts, floods, displacement, and weakened essential services repeatedly recreate the ecology of transmission [1], [2]. These same climate shocks also directly affect animal health and welfare: drought-related shortages of pasture and water can weaken livestock, increase mortality and disease vulnerability, reduce milk and meat production, and erode livestock assets on which pastoral households depend for food and income [3], [4]. Thus, climate-related disruption in Somalia affects not only human well-being but also the animals, livelihoods, and food systems that connect human and animal health.

The climate–outbreak connection in Somalia is mechanistic, not rhetorical. Climate shock can drive livelihood loss, livestock deaths, and water scarcity; these pressures force displacement into overcrowded settlements with unsafe water, inadequate sanitation, worsening undernutrition, and disrupted care. At the same time, deteriorating animal condition and reduced livestock productivity can undermine household food security and economic resilience, while concentration of people and animals around limited water and grazing resources may increase opportunities for disease transmission. In Somalia, only 52% of the population has access to basic water and 38% to basic sanitation, while drought concentrates communities around unsafe sources and floods breach latrines and contaminate shallow water systems. These interacting pressures contribute to recurrent AWD/cholera, measles, and other infectious threats without making climate change their sole cause. Rather, climate variability amplifies pre-existing fragility and creates conditions in which transmission becomes more likely and control becomes harder [5].

Somalia should therefore redefine health security as the capacity to prevent climate-related health emergencies, not merely to respond after outbreaks begin. Health sovereignty, in this operational sense, means the ability to generate, coordinate, finance, and act on Somali health intelligence across national and subnational levels while sustaining productive partnerships. It includes surveillance, laboratory capacity, community health workers, climate and early-warning information, WASH preparedness, local logistics, animal-health surveillance, environmental monitoring, and stronger domestic financing and workforce capacity. This is not a call for isolation from international assistance. It is a call to convert external support into durable national and community capability [1].

One Health provides the necessary architecture because Somalia's risks do not respect sectoral boundaries. Human disease surveillance alone cannot anticipate threats shaped by rainfall failure, river flooding, livestock stress, water contamination, malnutrition, and displacement. An integrated Somalia Climate–One Health Early Warning and Response Platform should therefore connect meteorological data, water availability, animal-health signals, human disease surveillance, malnutrition surveillance, displacement tracking, and WASH indicators. One Health surveillance is designed precisely to integrate human, animal, and environmental intelligence and, when it includes drivers of emergence rather than pathogens alone, can improve prevention, detection, and response. In Somalia, such a platform could identify high-risk districts early enough to trigger vaccination, chlorination, mobile outreach, nutrition support, livestock interventions, and targeted risk communication before outbreaks escalate [6].

Several practical steps follow. First, Somalia should establish a nationally coordinated Climate–One Health early-warning mechanism institutionalized within government rather than left as a sequence of short-term projects. Second, district laboratories and community-based surveillance should be strengthened so abnormal signals are detected before they become humanitarian emergencies. Third, climate-risk planning should integrate WASH, nutrition, vaccination, livestock health, and primary healthcare instead of treating them as parallel emergency sectors. Fourth, authorities and partners should pre-position medicines, vaccines, water-treatment supplies, and nutrition commodities ahead of predictable seasonal hazards, supported by pre-arranged climate-risk financing. Fifth, external assistance should be aligned with Somali leadership, workforce development, interoperable data systems, and durable institutional capacity rather than repeated stand-alone surge responses [7].

The cost of reactivity is both economic and epidemiological. Across 210 outbreaks in low- and middle-income countries, outbreak response immunization averted an estimated 5.81 million cases, 327,000 deaths, and US$31.7 billion in economic costs [8]. showing clearly that emergency response saves lives and must be preserved. But that same evidence also shows how costly repeated late action can become when systems wait for outbreaks to grow before mobilizing. The objective is not to eliminate emergency response, but to reduce the number, scale, and severity of emergencies requiring emergency response through preparedness, earlier detection, and anticipatory protection.

Somalia cannot outbreak-respond its way out of a climate crisis. The country must move from shock → outbreak → emergency response → temporary recovery → next shock toward risk prediction → prevention → resilient services → early action → rapid response → recovery and adaptation. One Health offers the institutional framework for that transition, and health sovereignty gives it a practical policy goal. Somali authorities, development partners, humanitarian agencies, researchers, and donors should now treat climate-resilient prevention capacity as a core investment in national health security. Somalia's strongest defense against climate-sensitive outbreaks is not faster response alone, but a health system able to anticipate and prevent the conditions that make outbreaks catastrophic.

## Clinical trial

Not applicable.

## CRediT authorship contribution statement

**Ayuub Ahmed Osman:** Supervision. **Maryan Ali Adam:** Investigation. **Abdisamad Abdihakim Aadam:** Conceptualization. **Ilyas Abdullahi Khalif:** Writing – review & editing. **Mohamed Abdijabar Mohamed:** Investigation. **Maryamo Maxamed Cali:** Supervision.

## Consent to participate

Not applicable.

## Consent to publish

Not applicable.

## Ethical approval

This article did not involve human participants, human data, or animals; therefore, ethical approval was not required.

## Funding

The authors received no specific funding for this work.

## Declaration of competing interest

The authors declare no competing interests.

## Data Availability

No data was used for the research described in the article.
